# Body weight algorithm predicts humane endpoint in an intracranial rat glioma model

**DOI:** 10.1038/s41598-020-65783-7

**Published:** 2020-06-02

**Authors:** Simeon O. A. Helgers, Steven R. Talbot, Ann-Kristin Riedesel, Laura Wassermann, Zhiqun Wu, Joachim K. Krauss, Christine Häger, André Bleich, Kerstin Schwabe

**Affiliations:** 1 Department of Neurosurgery, Hannover Medical School, Hannover, Lower Saxony Germany; 2 Institute for Laboratory Animal Science, Hannover Medical School, Hannover, Lower Saxony Germany

**Keywords:** Cancer models, CNS cancer, Cancer in the nervous system, Experimental models of disease

## Abstract

Humane endpoint determination is fundamental in animal experimentation. Despite commonly accepted endpoint criteria for intracranial tumour models (20% body weight loss and deteriorated clinical score) some animals still die before being euthanized in current research. We here systematically evaluated other measures as surrogates for a more reliable humane endpoint determination. Adult male BDIX rats (n = 119) with intracranial glioma formation after BT4Ca cell-injection were used. Clinical score and body weight were assessed daily. One subgroup (n = 14) was assessed daily for species-specific (nesting, burrowing), motor (distance, coordination) and social behaviour. Another subgroup (n = 8) was implanted with a telemetric device for monitoring heart rate (variability), temperature and activity. Body weight and clinical score of all other rats were used for training (n = 34) and validation (n = 63) of an elaborate body weight course analysis algorithm for endpoint detection. BT4Ca cell-injection reliably induced fast-growing tumours. No behavioural or physiological parameter detected deteriorations of the clinical state earlier or more reliable than clinical scoring by experienced observers. However, the body weight course analysis algorithm predicted endpoints in 97% of animals without confounding observer-dependent factors. Clinical scoring together with the novel algorithm enables highly reliable and observer-independent endpoint determination in a rodent intracranial tumour model.

## Introduction

In experimental rodent studies dealing with intracranial tumour formation, humane endpoints are often described in an imprecise way or even not mentioned at all, although survival rates are commonly used for Kaplan-Meier survival analysis. While in the early 90 s ‘death’ was used as endpoint criterion^1–3^, in more recent publications a weight-loss threshold of 20% has been suggested which, however, cannot be generalized across animal models^4^.

Furthermore, rather vague criteria have been used such as ‘rats were euthanized at any sign of poor health condition’^5^ or ‘rats were killed just before the expected date of death’^6^. Except for weight, however, most criteria that have been reported in this context are qualitative and highly observer-dependent^7^. Also, authors often do not differentiate whether rats were euthanized or died spontaneously^8,9^. The Council Directive 2010/63/EU requires that animals must be sacrificed at the earliest possible time point whenever death is foreseeable to reduce the duration and intensity of suffering.

Models of fast-growing intracranial tumours, such as gliomas, are especially demanding in the definition of humane endpoints. The progression of this disease is often characterized by a long asymptomatic phase despite the considerable dimension of the tumour, followed by a sudden and severe deterioration of the clinical condition^7,10^. This pattern is also seen in human patients^11^. Recently, we reported on an intracranial glioma-model which can be used to test the anti-tumour effects of local therapeutic strategies^10^. Remarkably, a 20% body weight-loss was never reached by any animal.

Within the frame of a national research consortium focusing on evidence-based severity assessment in different disease models, we set out to refine humane endpoints with objective and quantifiable criteria^12^. Measures of motor behaviour, species-specific behaviour and social interaction have recently been proposed to be useful for the assessment of well-being and humane endpoint determination^13–17^. Furthermore, monitoring of physiological markers, such as heart rate and body temperature, measured using telemetric methods have been considered to be valuable since it allows monitoring of animals without the presence of investigators in the vicinity of the animal^18^. A more elaborate analysis of the body weight course can also lead to strategies for a more objective definition of endpoint criteria. This has already been proposed but has not yet been applied with sufficient accuracy^3^.

In the current study, we used an intracranial glioma model to determine whether behavioural tests, as well as physiological markers, are useful in the definition of humane endpoint criteria and whether they are suitable to detect deteriorations of well-being before they become obvious by clinical scoring and weight-loss. Therefore, rats of the glioma-only group (n = 14, including 5 rats randomly chosen for tumour resection on the 8^th^ day after cell injection) were daily assessed for species-specific (nesting, burrowing), motor (distance, coordination) and social behaviour after tumour cell injection. Another group (n = 8) was subcutaneously implanted with a telemetric device four weeks before the cell injection for continuous monitoring of heart rate (variability), temperature and activity.

Furthermore, a novel algorithm for body weight course analyses is proposed, which can function as a warning system for endpoint determination. For its development, body weight and clinical scores of rats with intracranial glioma formation (n = 97), that had been used for systemic and local pharmacological studies before, were used.

## Results

### Postoperative outcome

Rats recovered after intracranial tumour cell injection within one day without relevant deterioration of clinical scores and weight-loss. No wound infections or neurological deficits were observed. Histological analysis showed tumour formation in all rats as reported in Wu *et al*. (2018; Suppl. Fig. 1). Survival times of the transmitter-implanted animals and the glioma-only animals were on average 15 days after tumour injection and that of the resection group was 12 days after tumour resection.

**Figure 1:**
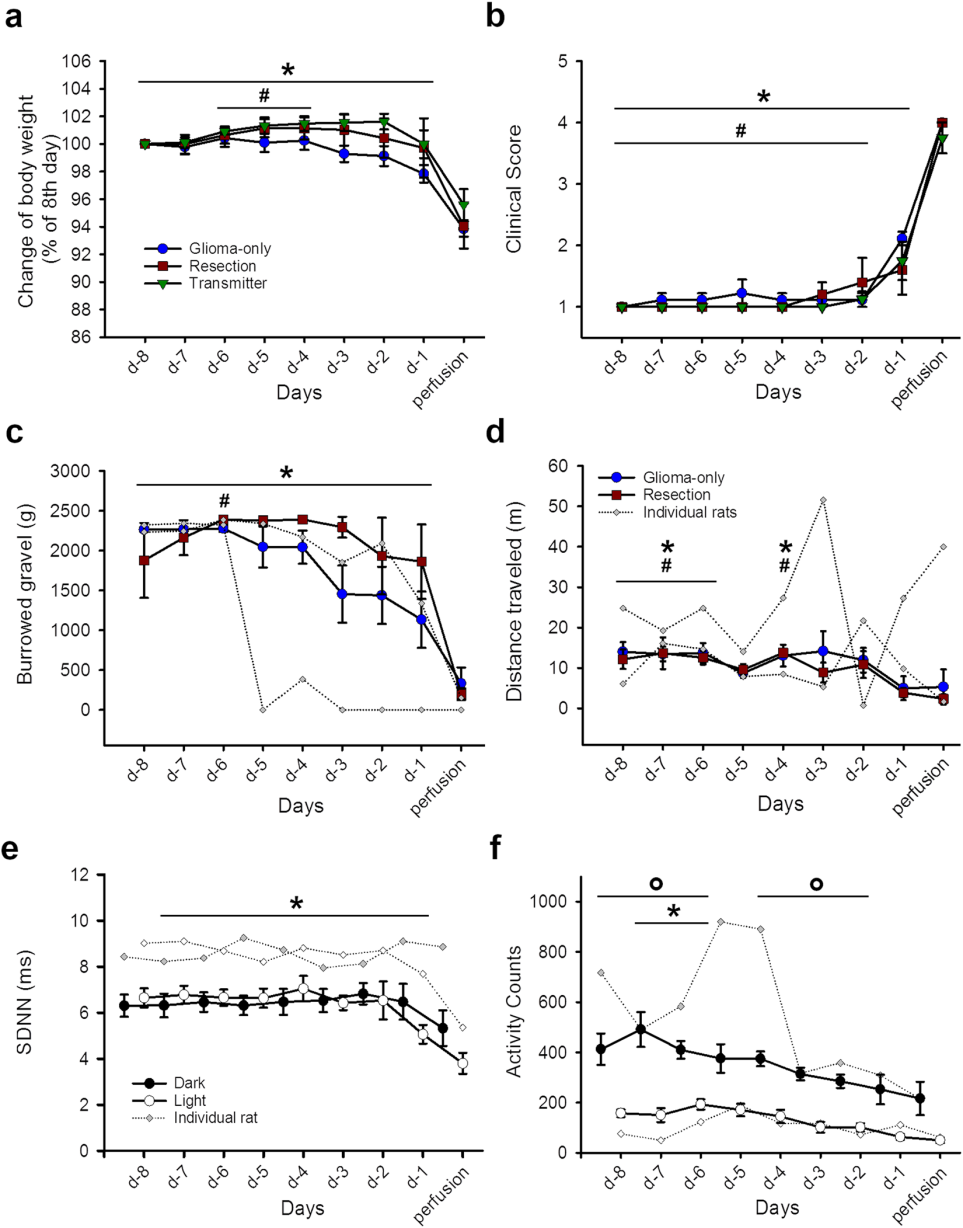
Physiological and behavioural parameters for the last eight days before endpoint. (**a**) Body weight change in per cent compared to d-8; significant differences were found between the day of the endpoint compared to all other days and between the second last day compared to day -6, -5 and -4 (F_(8,144)_ = 33.769, p < 0.001). (**b**) Clinical score was increased on the last two days compared to all previous days (F_(8,144)_ = 195,069, p < 0.001). (**c**) Burrowed gravel in gram (g) was decreased on the day of the endpoint compared to all other days and on the second last day compared to day -6 (F_(8,96)_ = 14.937, p < 0.001). (**d**) Distance travelled in meters (m) in the open field; significant differences were shown on day -8, -7, -6 and -4 compared to the last two days (F_(8,96)_ = 4.836, p < 0.001). Data are shown for the glioma-only and the resection group as mean ± S.E.M. Additionally, absolute values of two individual animals are shown with greyed symbols in c and d. (**e**) Total home cage activity measured by telemetric devices; significant differences were observed between dark and light phase except for day -5 (F_(1,48)_ = 173.271, p < 0.001) and between day -7 and -6 compared to the last day (F_(8,48)_ = 6.268, p < 0.001). (**f**) Heart rate variability in milliseconds (ms) was decreased the last day compared to all previous days except for day-2 (F_(8,48)_ = 4.680, p < 0.001). Data are shown for the central 8 hours of the dark and the light phase measured by the telemetric device as mean ± S.E.M. Absolut values of one individual animal are shown with grey symbols in e and f. Significant differences compared to the day of the perfusion are shown as asterisks (*), differences compared to d-1 are shown as hashtags (#) and differences between the dark and the light phase are shown as circles (o). Two-way RM ANOVA with a post-hoc test (p < 0.05).

One rat of the transmitter group died without being euthanized. Another rat was considerably older and heavier than the other animals of the group. Physiological parameters of this rat differed from all other tested animals and were therefore excluded from the analysis but shown in the graphs as “greyed” values (Fig. 1e,f).

### Body weight/clinical score

Body weight and clinical scores were stable until rats showed a rapid body weight-loss and a deteriorated clinical score on the last day (Fig. 1a,b). Clinical scores also significantly deteriorated on the second last day before the endpoint.

### Behavioural testing

Rats of the glioma-only and the resection group burrowed less material towards the endpoint (Fig. 1c). Post-hoc testing showed that burrowing decreased on the day of the endpoint compared to all other days. Instead, nest complexity was unaffected towards the endpoint formation (Suppl. Fig. 2a).

The duration and the frequency of social interactions were not altered towards the endpoint (Suppl. Fig. 2b, c). More detailed analysis of the parameters playing, following, anogenital sniffing and head sniffing showed also no effect.

General motor activity, measured by the total distance travelled in the open field, was decreased on the last two days compared to the days -8, -7, -6 and -4 (Fig. 1d). Analysis of the motor coordination on the balance beam showed that rats were slower on the last days before the endpoint (Suppl. Fig. 2d). Furthermore, rats were more deteriorated after tumour resection, but not towards the endpoint. For the rotarod, deterioration was only shown between the day of the endpoint and day -4 and -5 (Suppl. Fig. 2e).

### Telemetric measurements

Rats show a clear circadian rhythm with variability of heart rate between dark and light phase. This circadian rhythm broke down on the last two days before the endpoint (Suppl. Fig. 3). The **standard deviation of normal-to-normal [heart beat] intervals (SDNN)** decreased towards the end of the experiment compared to most other days (Fig. 1e). In contrast to the heart rate, no differences between the dark and light phase were observed. The body temperature of the animals was mostly stable throughout the whole experiment with a clear circadian dark/light rhythm (Suppl. Fig. 2). Activity, measured by the implanted telemetric device, showed gradually decreasing activity counts towards the end of the experiment together with a clear dark/light difference (Fig. 1f).

### Deterioration of behavioural and physiological measures on an individual level on the last day

Body weight and clinical score, burrowing behaviour, balance beam performance and the SDNN during the light phase significantly decreased on the last day (endpoint) compared to the previous day. Remarkably, only for body weight, clinical score and SDNN 100% of individual animals paralleled the effects observed on a group level. In all other tests, only parts of the animals followed the effects observed on a group level and are therefore regarded as not reliable for humane endpoint determination (Table 1).

**Table 1:** Number of individual rats that deteriorate on the day of the endpoint.

	Endpoint
*Body weight loss*	22/22*
*Deteriorated clinical score*	22/22*
**Species-specific behaviour**	
*Decreased nest complexity*	3/9
*Less Burrowing*	9/14*
**Motor related**	
*Decreased distance*	10/14
*Slower on balance-beam*	10/14*
*Shorter time on rotarod*	8/9
**Social behaviour**	
*Decreased total interaction (Duration)*	6/8
*Decreased total interaction (Frequency)*	6/8
**Telemetry**	
*Increased heart rate (Light)*	4/7
*Decreased heart rate(Dark)*	4/7
*Decreased SDNN (Light)*	7/7*
*Decreased SDNN (Dark)*	6/7
*Decreased activity (Light)*	5/7
*Decreased activity (Dark)*	6/7
*Increased body temperature (Light)*	4/7
*Decreased body temperature (Dark)*	4/7

SDNN = standard deviation of the heartbeat intervals; *significant differences observed on group level between the second last and the last day.

### Principal component analysis

Principal component analysis (PCA) reinforced our previous assumption that despite voluminous intracranial tumour formation, the overall rat condition did not deteriorate until one day before the endpoint. Also, clinical scores, body weight change and SDNN were identified as important components.

**Figure 2:**
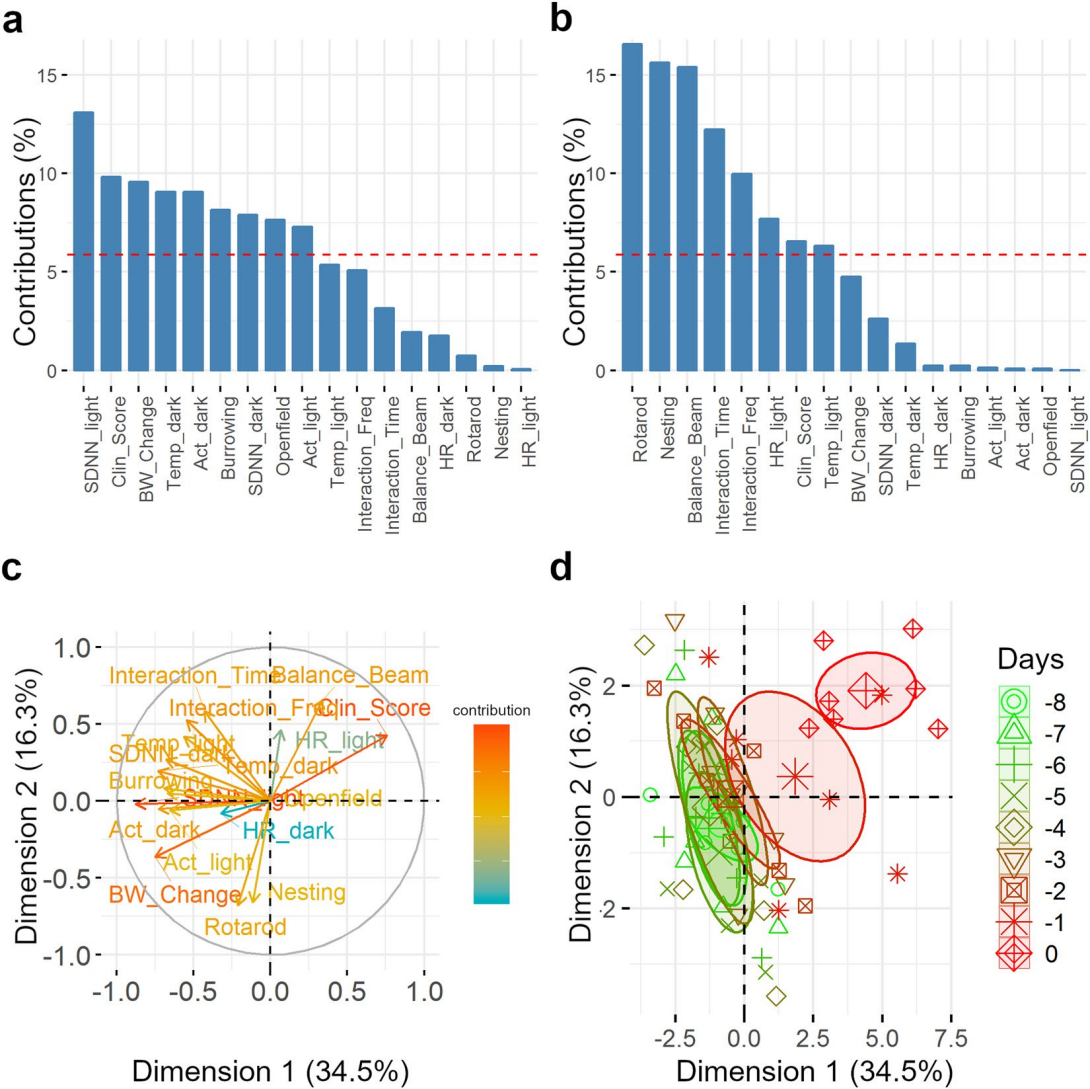
Principal component analysis of behavioural and physiological parameters for the last 8 days before the endpoint. Contribution of the different parameters to the first dimension (**a**) and the second dimension (**b**) are shown in per cent. The dashed line indicates the threshold for the equal contribution of each parameter. Illustration of the first two dimensions of the principal component analysis is shown as arrows (**c**). Length, direction and colour of the arrows code for the level of contribution of each parameter to the different dimensions. Clusters are based on the principal component analysis for each day (**d**). 95% confidence ellipses around group clusters (days) are shown as different colours and symbols for each day. BW_Change – body weight change (%); Clin_Score – clinical score; HR_dark – heart rate (dark phase, bpm); SDNN_dark – heart rate variability (dark phase, ms); Act_dark – activity counts (dark phase); Temp_dark – body temperature (dark phase, °C); HR_light – heart rate (light phase, bpm); SDNN_light – heart rate variability (light phase, ms); Act_light – activity counts (light phase); Temp_light – body temperature (light phase, °C); Burrowing – burrowed gravel (g); Openfield – distance traveled in the open field (m); Nesting – nest complexity; Balance_Beam – completion time of the balance beam test (sec); Rotarod – time until falling form the rod (sec); Interaction_Time – total interaction time (sec); Interaction_Freq – interaction frequency.

In more detail, PCA of the pooled variables for day -8 until the endpoint revealed that 34.5% of the variation could be explained with the first dimension, whereas the second dimension explained 16.3% (Fig. 2c). The first principal component was dominated by the SDNN during the light phase. Furthermore, clinical scores and body weight change largely contributed to this dimension (Fig. 2a). The second dimension, however, was dominated by the parameters of the motor coordination tests and nesting behaviour (Fig. 2b). Confidence ellipses (95%) around group clusters (days) allow distinguishing the endpoint from all other days. This endpoint, however, was already announced by an incomplete separation of the day before (Fig. 2d).

### Endpoint detection algorithm

**Figure 3:**
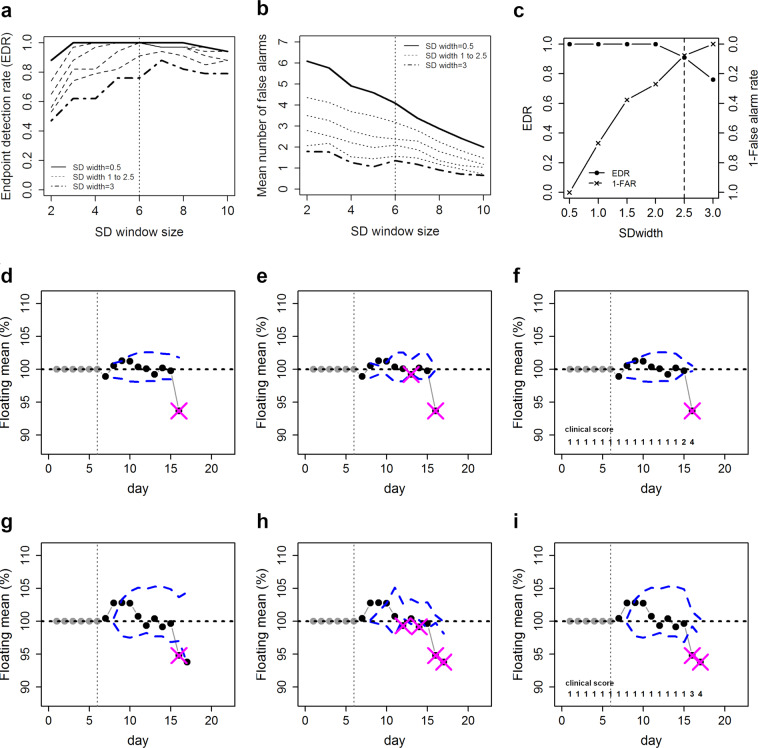
Performance and evaluation of endpoint detection algorithm. (**a**) Endpoint detection rate and (**b**) mean number of false alarms per animal for different SD window sizes and SD widths. The optimal window size is indicated by a vertical dashed line. Endpoint detection rate and false alarm rate at the optimal window size of 6 days for different SD widths (**c**). Optimal SD width is indicated by the dashed line. Endpoint detection using normalized body weight change data with optimized settings (SD window size = 6, SD width = 2.5) in the regular mode (**d**,**g**), the MAD constrained mode (**e**,**g**) and score constrained mode (**f**,**i**) for two individual animals (**d**–**f** and **g**–**i**). Boundaries are indicated by the dashed lines and alarms by the crosses.

Although PCA clearly showed a deterioration of the animal’s condition on the day of the endpoint on a group level, this strategy does not allow endpoint detection on an individual level. In our rat glioblastoma model, behavioural tests and telemetric measurements were not superior to weight and clinical scores as humane endpoint criterion. Therefore, we focused on measuring body weight as an objective parameter to develop an endpoint detection algorithm. Based on the body weight course, the algorithm defines boundaries covering the physiological body weight changes of each animal on each day. Whenever the lower boundary is violated by a reduced body weight measurement on a given day, the experimenter is warned that the animal is ‘at risk’ (Fig. 3d–i, pink crosses).

In more detail, for each consecutive day and animal, the algorithm calculates the unweighted mean (moving average) of a specific number of previous days (window size). Boundaries (Fig. 3d–i, blue dashed lines) around the moving average were determined by multiplying the standard deviation (SD) of the moving average with a specific width-factor. To enhance the sensitivity for boundary violation by abnormal weight reduction, the algorithm can be used in different modes: (1) regular, i.e., without constraints, as well as boundaries narrowed by either (2) the mean absolute deviation (MAD), i.e., the mean of the lagging data window minus the observed data point, or (3) the clinical score. If the warning occurs at the actual endpoint, it was counted as a true positive event (correct warning), otherwise as false positive (false alarm). For a more detailed description see the Methods section (Table 2 for definitions).

**Table 2:** Definitions of terms used for the description of the endpoint detection algorithm.

Moving average	Unweighted mean of the previous number of data points; here: average body weight of a certain number of previous days
Window size	number of previous data points; here: number of days used for calculation of the moving average
Width-factor	standard deviation multiplication factor; here: for the calculation of the boundary widths
Boundaries	upper and lower decision levels around the moving average; here: calculated by SD multiplied by the width factor
MAD	mean absolute deviation; here: mean of the lagging data window minus the observed data point
EDR	endpoint detection rate; here: the proportion of correctly identified endpoints

For the optimization of the algorithm, the window size (2–10 days) and width-factor (0.5–3) was varied and evaluated on a subsample of the whole data set, i.e., the training data, as a proof of concept. Here, the endpoint detection rate (EDR; the proportion of correct warnings) and the mean number of false alarms per animal (Fig. 3a,b) were used to evaluate the performance of the algorithm. When using width-factors of 0.5–1.5, the algorithm correctly identified all endpoints, however, at the cost of an increased number of false alarms (Fig. 3a,b). Importantly, the number of false alarms depended more on the width-factor than on the window size, which had an optimum at 6 days. With a window size set to 6 days, the optimal width-factor was determined (Fig. 3c). Based on the EDR and the false alarm rate the optimal width-factor was determined to be 2.5.

The performance of the algorithm with the optimized parameters in each mode for the training set is shown in Table 3. In the training data, the regular run resulted in an EDR of 91% with 1.56 false alarms per animal. In three animals the endpoint was not correctly identified. In the MAD and score constrained modes, all endpoints were identified correctly, however with a slightly increased number of false alarms.

**Table 3:** Performance of the endpoint detection algorithm in all three modes on the training and the validation set.

	Training set			Validation set		
	regular	MAD	score	regular	MAD	score
set size (n)	34	34	34	63	63	63
failed detections	3	0	0	11	2	2
mean # false alarms	1.56	2.76	1.97	1.13	2.63	1.43
EDR	0.91	1	1	0.83	0.97	0.97

EDR = endpoint detection rate; MAD = mean absolute deviation.

The algorithm was then tested retrospectively on the validation data set (Table 3). In the regular mode, the algorithm gave an EDR of 83%, which substantially improved in the MAD and the score constrained mode. In both modes, only two animals were not classified correctly (EDR: 97%). The number of false alarms was substantially increased in the MAD constrained mode compared to the score constrained mode. In the MAD and the score constrained mode, the algorithm even detected the 6 rats in the training and validation set, for which the original endpoint criteria failed. In the regular mode, the endpoint algorithm failed only in one of those animals.

Regarding the different modes, it should be noted that in rats with stable body weight, all three modes correctly identify the endpoint (Fig. 3d–f). High variance in body weight, however, lead to wider boundaries so that endpoints were only correctly identified in the MAD and score-constrained mode, which in turn increased the number of false alarms (Fig. 3g–i).

## Discussion

Over the past few years, marked progress has been made in the understanding of neurobiology and the treatment of glial tumours^19–21^. Nevertheless, animal models still play an important role in advancing knowledge and improving treatment^22–25^. Public discussions on animal welfare, however, have caused the implementation of laws and guidelines that increasingly regulate such research^26,27^.

In clinical cancer trials, the Kaplan-Meier estimate is used to demonstrate survival functions, i.e., the fraction of patients living for a certain period after treatment. In experimental research, however, death as an intentional endpoint is legally and ethically unacceptable^7,28^. Nevertheless, despite reporting and defining endpoint criteria, the number of rats which accidentally die before reaching the endpoint criteria is often not reported^8,9,29^.

The major challenge of humane endpoint determination is that it is insufficient to have valid results across a group of animals, but that it has to be reliable for each animal. In the current study, we showed that behavioural tests either paralleled the severe deterioration of body weight and the clinical score 1–2 days before the endpoint or showed no deviation towards the endpoint at all. Notably, all rats lost weight in parallel with the deterioration of clinical scores on the day of the endpoint. Also, the SDNN deteriorated in all rats. Instead, although burrowing and motor coordination significantly deteriorated on a group level, this was not the case for all individual rats. Remarkably, elaborated body weight course analysis provided an objective, reliable warning system for humane endpoint determination even without reaching a 20% weight-loss, which is usually used as a criterion in experimental rodent studies.

In more detail, species-specific behaviour has been successfully applied for severity assessment of experimental procedures in mouse models^30–32^, but less often in rat models^33^. Its potential use as a criterion for the endpoint determination has not been tested so far. In the current study, on the individual level, only about two-thirds of the animals reliably showed a decline in burrowed gravel towards the endpoint. Nest complexity behaviour also showed no deterioration towards the endpoint at all but varied to a considerable extent from day-to-day.

Social interaction has been primarily reported to be beneficial for the well-being of rats in general and during experimental procedures^34^ and more recently social interaction has been suggested to be a useful parameter for severity assessment in epilepsy models^33^. Nevertheless, social interaction analysed in the current study was not altered towards the endpoint.

Concerning motor behaviour, the distance moved in the open field was stable even on the last day, whereas more detailed analysis of motor coordination showed significant deterioration on the day of the endpoint. However, on the rotarod, some animals learned to deliberately jump off the rotating rod. Likewise, when tested on the balance beam, some rats did not walk down the beam despite being able to, as shown by successful performance on the next day. Therefore, with these tests, it is difficult to differentiate whether rats are not able to perform, or not willing to perform.

One reason for the high variation could be that the experimenter influences the animal’s behaviour^35^. With that regard, telemetric measurements allow continuous control of activity and physiological parameters in the home cage without the presence of an experimenter^18,36,37^. Indeed, general motor activity measured with the telemetric device in the home cage slowly deteriorated during the last 8 days towards the endpoint. However, no clear drop was seen on the last 1–2 days, which make this measure unsuitable for endpoint determination.

While heart rate and temperature only deteriorated towards the endpoint to some extent, SDNN significantly deteriorated in 100% of the rats during the light phase, i.e., was equally suitable for endpoint detection as weight and clinical scores. Decreased SDNN was previously reported in relation to decreased well-being after surgery in mice^18^. Interestingly, except for the SDNN, all physiological parameters and the activity showed a clear circadian rhythm, which breaks down 1–2 days before the endpoint in case of heart rate and temperature. Notably, as already reported in previous studies, telemetric measurements are highly depended on animal age^38,39^. One animal implanted with a telemetric device was older and heavier than all other animals. All parameters measured for this rat considerably differed to the other rats. Therefore, the definition of endpoint criteria based on these measurements would be difficult to apply to rats of different ages.

Together, all behavioural and physiological measures support our assumption that despite considerable tumour dimensions, rats with intracranial tumour formation are in good condition until 1–2 days before the endpoint, which was also supported by PCA. Even rats of the resection group did not substantially differ from rats of the glioma-only group, although these rats were exposed to a second surgery involving a large craniotomy of the skull.

Slight deteriorations of the clinical score need experienced observers to reliably detect differences between score 1 and 2, as seen 2 days before endpoint. However, objective criteria for endpoint determination like a weight-loss threshold (e.g. 20%), also imposes problems as the extent of the final weight-loss differs across rats. Especially in young rats, which gain weight during development, this criterion is not applicable as it is highly dependent on the baseline.

Our algorithm reliably predicts the endpoint in up to 97% of all tested animals. It defines an individual threshold for each day and animal-based on the body weight course of the same animals on the previous days. Because of its flexibility, the algorithm is independent of the absolute body weight and the level of fluctuation and is, therefore, more reliable as endpoint criterion than behavioural and telemetric measurements. Notably, in all cases in which the previous endpoint criterion failed, the experimenter would have been warned on the last day the animal was alive. A drawback of the algorithm, however, is the relatively high number of false-positive alarms. Closer inspection of the false alarms shows that a substantial number of false alarms occur either on the day before the actual endpoint or on the day after tumour resection as second operation (adjusted number of mean false alarms: 0.83). Therefore, these false alarms can be classified as a correct indication of days where special attention is needed. It has to be noted that the algorithm should be used as an objective parameter to support the judging of the experimenter and to trigger closer inspection.

Together, all our experiments showed that the animals were in good health after the tumour cell injection and not affected by the intracranial tumour development until the endpoint. We showed that sophisticated body weight course analysis results in an objective, reliable and easy-to-use parameter for endpoint determination. This algorithm, in connection with clinical scoring, was shown to be more reliable than behavioural tests and telemetric measurements and may be translated to other models as well.

## Methods

### Animals

Adult male BDIX rats (>200 g, bred in the Central Animal Laboratory of Hannover Medical School) were housed in groups of two to four animals per Macrolon Type IV open cages under controlled environmental conditions (22 ± 2 °C, 55 ± 10% humidity) with a 14 h light - 10 h dark cycle (lights on at 6 am). Animals received normal diet (1324 TPF from Altromin Spezialfutter GmbH&Co. KG, Lage, Germany) and tap water ad libitum. Wood chip bedding material was provided (Espentiereinstreu AB P3, AsBe-wood GmbH; Gransee, Germany). All experiments were carried out following the EU directive 2010/63 and were approved by the local animal ethics committee (Lower Saxony State Office for Consumer Protection and Food Safety). All efforts were made to minimize pain or discomfort of the animals used.

### Study design

The data of 119 adult male BDIX rats with intracranial glioblastoma formation after BT4Ca cell injection were used for this study. Clinical scores and body weight were measured daily in all animals.

For behavioural assessment, one subgroup (n = 14; including 5 rats randomly chosen for tumour resection on the 8th day after cell injection) was daily assessed for species-specific (nesting, burrowing), motor (distance, coordination) and social behaviour between 9 am and 2 pm until endpoint criterion. Another subgroup (n = 8) was subcutaneously implanted with a telemetric device four weeks before the cell injection for continuous monitoring heart rate (variability), temperature and activity. Animals were euthanized and transcardially perfused when reaching the endpoint of deteriorated clinical score and weight-loss. Tumour growth was confirmed by microscopic analysis of Nissl-stained coronal brain slices.

For the development of an elaborate body weight course analysis algorithm for endpoint detection, body weight and clinical scores of rats with intracranial glioma formation were used, that had been used for systemic and local pharmacological studies before^4,10^. These rats were used for training (n = 36) and validation (n = 61) of the algorithm. Notably, in these sets, 6 rats had unexpectedly died without predication by previous endpoint criterion determination.

### Surgeries

All surgeries were performed under general chloral hydrate anaesthesia (360 mg/kg) with local anaesthesia and systemic analgesia with carprofen (5 mg/kg intraoperatively, 2.5 mg/kg postoperatively for two days). Tumour cell suspensions were stereotaxically injected through a burr hole. For tumour resection, a craniotomy was made and the tumour tissue was removed using microsurgical techniques (see Wu *et al*., 2018). The telemetric device (ETA F10; PhysioTel Telemetry System DSI; St Paul, MN, USA) was implanted subcutaneously and the ECG electrodes were tunnelled to the chest wall. For detailed information about surgical procedures see Supplementary information (Suppl.).

### Behavioural testing

Body weight and general health scores were assessed daily from tumour cell injection until the endpoint. For species-specific behaviour, the amount of gravel burrowed from a hollow tube within one hour, as well as nest-building with 14 g EnviroDry® scored with a nest complexity score was assessed. For general motor behaviour, the distance travelled within 10 minutes in an open field environment was measured. Motor coordination was assessed by the duration the animals spent on an accelerating rotarod and the time to walk 150 cm on a balance beam. Social interactions between two rats were filmed and the duration and frequency of interactions assessed. For detailed information about the behavioural tests see Suppl.

### Telemetric measurements

Physiological and activity data were monitored continuously immediately after tumour cell implantation until the endpoint was reached and animals were euthanized. For analysis of the transmitter data heart rate, standard deviations of the heart beat intervals (SDNN), temperature and activity were averaged and summed over 8 hours in the light (9 am–5 pm) and the dark phase (9 pm–5 am).

### Statistics

Statistical analysis was performed with SigmaStat software (SigmaStat 4.0, Systat Software Inc., 2016). Data of behavioural testing of the last 8 days before an endpoint were compared by two-way repeated-measures ANOVA with glioma-only and resection as factor groups and days as levels. For post-hoc analysis Tukey’s test was used and p < 0.05 considered significant. Four rats were not behaviourally tested on the last day because of severely deteriorated clinical scores. For these rats, the missing value was replaced with the worst value of the other rats on the last day. Values of telemetric measures were compared by two-way repeated-measures ANOVA with day and dark/light phase as factors. Post-hoc testing was performed using multiple Bonferroni-corrected paired t-tests.

For the endpoint determination, differences to the previous day on the level of the individual animal are especially important. Therefore, deterioration of behavioural and telemetric measures on the last day was counted for each individual rat.

Graphs of behavioural and telemetric measures were plotted with SigmaPlot software (SigmaPlot 14.0, Systat Software Inc., 2017). All measurements are shown as mean ± SEM. Values of individual animals are shown in the graphs of the behavioural tests to highlight strong deviation from the mean.

Also, principal component analysis (PCA) was conducted using the factoextra^40^ package in base R^41^. PCA requests complete data sets, but behavioural and telemetric data were achieved from two different rat subgroups. Therefore, normalized body weight on the last day of the transmitter group and the glioma-only group was ranked and two rats (one of each group) were matched and pooled for the PCA according to their rank. All behavioural and physiological parameters were included in the analysis. The principal components of the first two dimensions for all days were plotted as well as the factor loadings and contributions.

### Endpoint detection algorithm

Body weight was normalized to 100% (starting value). For each time point (days) the mean and standard deviation (SD) was calculated for a defined window of days preceding the current time point. This moving average gives only results for the days following the defined window size. Boundaries around data points were calculated by multiplying the SD with different width-factors ranging from 0.5–3 and adding/subtracting these from the moving average. Data points violating the lower boundary indicate potential danger to the animal. False alarms occur when the boundaries are violated but the actual endpoint is not reached.

The endpoint detection algorithm can be run in three modes. 1. Regular run with no constraints. 2. Run constrained by the mean absolute deviation (MAD) of the current data points. For this, the boundaries are locally narrowed by the rounded MAD value. 3. Run constrained by the clinical score. Here, the boundaries are constrained by actual clinical scores. The algorithm was evaluated with a training (n = 34) and validation set (n = 63).

A graphical user interface for the endpoint algorithm (running the SD model) is available as a Shiny-App under the following link: https://calliope.shinyapps.io/endpointer. Here, users can upload data for endpoint evaluation. Since the application runs on external commercial servers, the authors do not accept any liabilities. Also, by clicking on the link the authors cannot guarantee conformity with the terms of the General Data Protection Regulation GDPR of the European Union.

## Supplementary information

Supplementary Information.

## Data Availability

Raw data will be provided upon request.
