# Supplementary material for: Body weight algorithm predicts humane endpoint in an intracranial rat glioma model
Source: Sci Rep. 2020 Jun 2;10:9020. doi: 10.1038/s41598-020-65783-7 (PMC7265476; doi:10.1038/s41598-020-65783-7)
Supplement: Supplementary file 1 — Supplementary Information. [file 41598_2020_65783_MOESM1_ESM.pdf]

Body weight algorithm predicts humane endpoint in an intracranial rat glioma model

Simeon O.A. Helgers[1*#] MSc, *Steven R. Talbot*[2#] *Dr. rer. nat.*, *Ann-Kristin Riedesel*[1], *Laura Wassermann*[2], *Zhiqun Wu*[1] *M.D.*, *Joachim K. Krauss*[1] M.D., *Christine Häger*[2] *Dr. rer. nat.*, *André Bleich*[2] PhD, *Kerstin Schwabe*[1] PhD

[1]*Department of Neurosurgery, Hannover Medical School, Hannover, Lower Saxony, Germany*
[2]*Institute for Laboratory Animal Science, Hannover Medical School, Hannover, Lower Saxony, Germany*
[#]*contributed equally*

Supplementary Information

Materials and Methods

BT4Ca cell injection and tumor resection

Rats were anesthetized with 360mg/kg chloral hydrate via intraperitoneal injection (Sigma-Aldrich Chemie GmbH, Steinheim, Germany). Additionally, 2% xylocaine (AstraZeneca GmbH, Wedel, Germany) was used as local anesthetic. For analgesia Carprofen (RIMADYL®, Pfizer GmbH, Berlin, Germany) was injected subcutaneously with 5mg/kg intraoperatively and 2.5mg/kg for the first two postoperative days. The rat was fixed in a stereotaxic frame and the skin was opened with a medial cut. Bregma was used as reference point for the target coordinates +2.6mm in the anterior/posterior direction and +2.5mm in the lateral direction. A small burr hole was drilled at these coordinates. With a Hamilton syringe (SEG Analytical Science Pry. Ltd., Victoria, Australia) a cell suspension of 104 BT4Ca cells in 3µl PBS was injected at a depth of 2.8mm with a velocity of 1µl/min. For tumor resection 8 days after cell injection general anesthesia and analgesia was induced as described for tumor cell injection. Rats of the resection subgroup were fixed in the stereotaxic frame and their midline wounds were reopened. A craniotomy was made extending approximately 2mm radially from the original burr hole with a hand-held drill. After opening of the dura, the arachnoid was incised and the tumor was resected using microsurgical techniques. Thereafter the skin incision was closed with suture clips (see Wu et al., 2018).

Transmitter implantation

General anesthesia and analgesia was induced as described for the tumor cell injection. A 2cm long incision was made lateral to the spine and caudal to the left costal arch. Subcutaneous tissue was bluntly dissected and the telemetric device (ETA F10; PhysioTel Telemetry System DSI; St Paul, MN, USA) was placed into a previous prepared cavity. The ECG electrodes were tunneled. The negative electrode was embedded in the right pectoralis major muscle and the positive electrode was placed approximately 1cm lateral to the left side of the xiphoid process. Both electrodes were fixed with non-absorbable suture material. Incisions were closed with sutures and suture clips. Data was obtained by Ponemah 6.41 (PhysioTel Telemetry System DSI; St Paul, MN, USA).

Monitoring of body weights and clinical scoring

Body weight and general health scores were assessed on a daily basis from tumor cell injection until the endpoint. The health score was assessed according to the following grading: 1 – active, strong and fast movements, normal exploratory behavior; 2 – active with some interruptions of activity; exploratory behavior; 3 – prolonged lack of activity, limited exploratory behavior upon external stimuli, intact muscle tonus; 4 – ataxia, no exploratory behavior, severely limited reaction to external stimuli, decreased muscle tone, hunched posture, ruffled fur, delayed righting reflex; 5 – no righting reflex, premortal stage. Whenever an animal reached a score of 3, the animal was controlled twice a day. At scores 4 the animal was euthanized, as the death of the animal was expected within the next hours.

Behavioral testing

Burrowing

Burrowing behavior was tested with tubes made out of grey plastic with a sealed backside and an elevated open front side (length: 320mm, diameter: 100mm, elevation of front side: 60mm) as described in Deacon (2006). The tube was filled with 2.5kg of gravel (2-4mm diameter). After each trial the gravel was cleaned in 0.1% acetic acid. Prior to surgery the animals were habituated for one day to the test environment by placing them for one hour in a Macrolon Type IV cage with an empty burrowing tube. For testing the animals were placed into an empty cage for 30 minutes; afterwards the gravel filled tube was placed for one hour in their cages. After completion, the amount of gravel which was burrowed out of the tube was weighted.

Nest building

Rats received 14g Enviro-dri® (Claus GmbH, Limburgerhof, Germany) per animal once weekly upon cage cleaning. Every morning photos of the nest were taken. These photos were then arranged as one photo assembly for each cage and day. Two raters scored the photo assemblies after masked randomization according to a modified version of the scoring system proposed by Van Loo and Baumans (2004) and Schwabe et al., (2019 – submitted). For evaluation the mean score across the raters was used.

Social interaction

Social interaction was tested by placing rats together with an age-matched social interaction cage partner in an open field environment. Social interaction was filmed for 5 minutes from above the box and analyzed according to four different parameters: playing, following, anogenital sniffing and sniffing at the head. For each parameter the frequency and the duration of the behavior were assessed.

Locomotion

Locomotive activity was tested in an open field environment (62x62x30cm). The rats were habituated to the box on the day before the tumor cell injection for 10 minutes. For experimental testing the rat was placed in the middle of the open field. Its locomotive activity was recorded for 10 minutes by a video camera located above the box. The total distance travelled was analyzed online using TopScan (TopView Analyzing System 2.0; Clever Sys Inc.).

Motor coordination and balance

For assessment of motor coordination and balance the animals were tested in a rotarod chamber (10.5cm × 43cm × 43cm, Rotarod, series 8, IITC Life Science). On the day prior to surgery all animals were habituated to the chamber. The starting speed of the rod was five rotations per minute (rpm) accelerating up to 15rpm within 60 seconds, followed by another 60 seconds with constant 15rpm. For testing, the animals were placed onto the rod for three consecutive trials. The trials were terminated

when the rat fell of the rod, or when the animal completed the whole 120 seconds test duration. The mean values of one block consisting of three trials were used for statistical analysis.

Since several rats learned to deliberately jump off the rotating rod, the balance beam test was used additionally. This test is performed on a quadratic wooden bar (length: 1500mm, diameter 18mm x 18mm), which was installed in a height of 400mm with a platform at each side of the bar. On the end platform reward pellets (Dustless Precision Pellets®, Rodent Purified Diet; BioServ, Flemington, USA) were placed and the home cage was placed directly behind the end platform to encourage rats to walk down the bar.

Prior to the surgery the animals were habituated to the test environment and trained to walk over the beam to collect the reward. During the testing phase the rats were placed on the start position and the time to reach the endpoint was measured. If the animal failed to reach the end platform within 60 seconds or fell of the beam, the run was terminated and 60 seconds was noted as the maximum time.

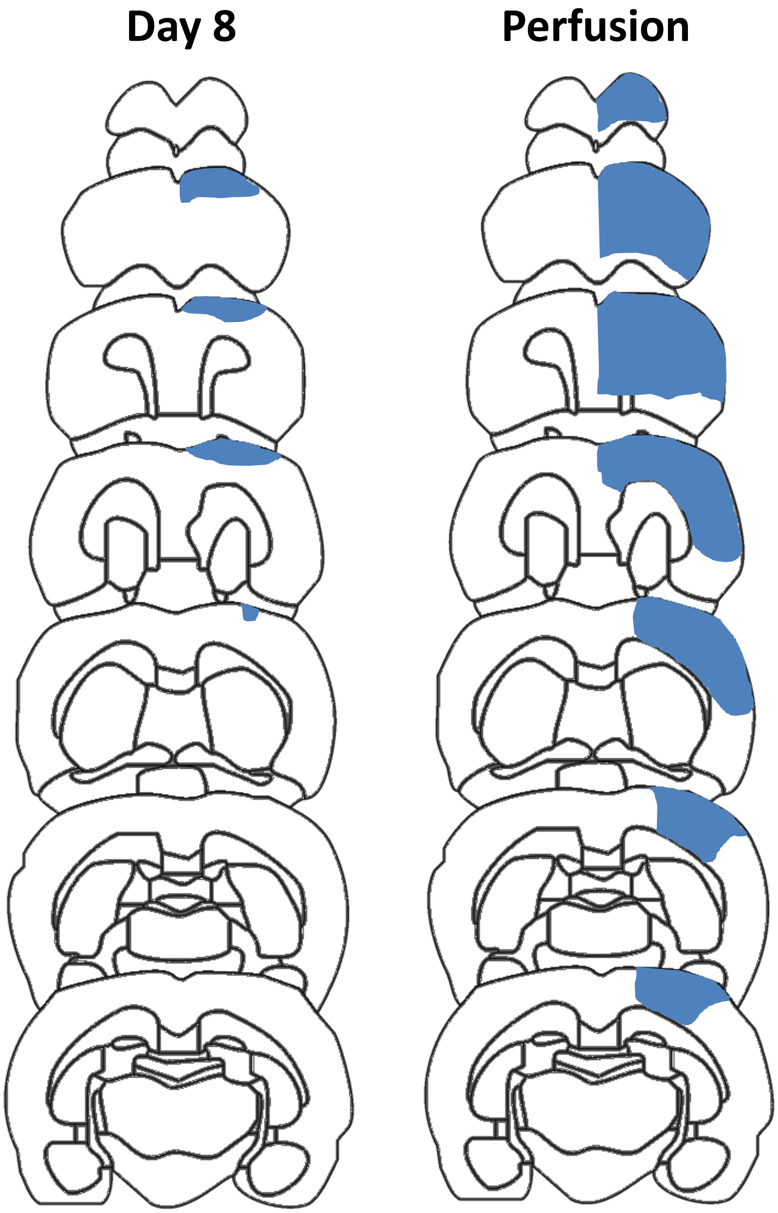

Suppl. Figure 1: Tumor dimension on day 8 after tumor cell injection (left) and on the final day (right) in a schematic drawing of coronal brain sections (adapted to Rumple, A. et al. 3-Dimensional Diffusion Tensor Imaging (DTI) Atlas of the Rat Brain. PLoS One 8, e67334 (2013))

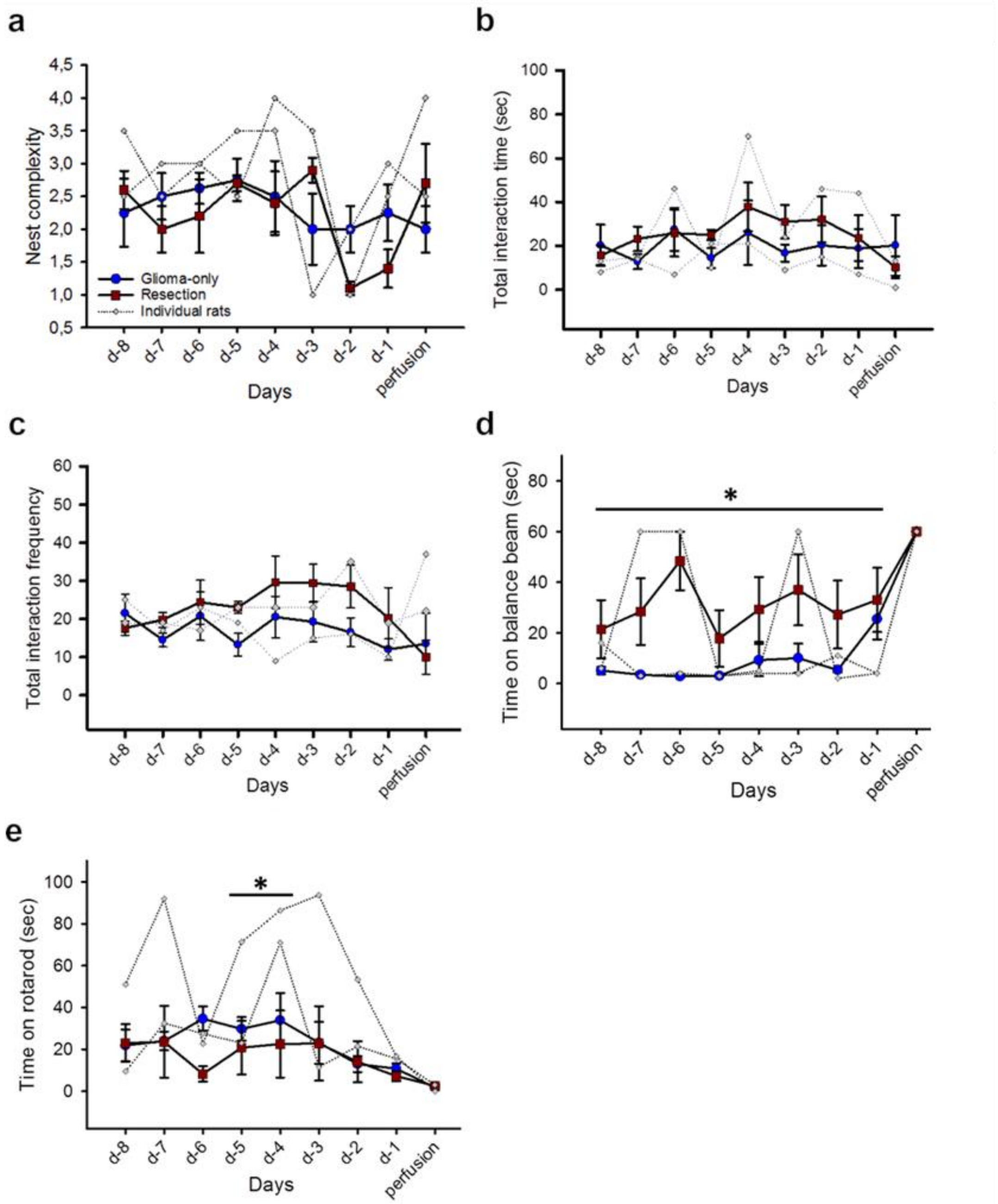

Suppl. Figure 2: (A) Nest complexity score. No significant differences were observed. (B) Total social interaction time (sec) and (C) total interaction frequency were not significantly altered. (D) Competition time on the balance beam (sec) was increased on the last day compared to all previous days ($F_{(8,96)}$=10.567, p<0.001). The experimental groups were shown to be significantly different ($F_{(1,96)}$=14.016, p=0.003). (E) Rotarod performance (sec) was significantly decreased on the last day compared to day -5 and -4 ($F_{(8,56)}$=3.099, p=0.006). Data are shown for the glioma-only and the resection group as mean ± S.E.M. Additionally, absolute values of two individual animals are shown with grayed symbols. Significant differences compared to the day of the perfusion are shown as asterisks (*). Two-way RM ANOVA with post-hoc test (p<0.05).

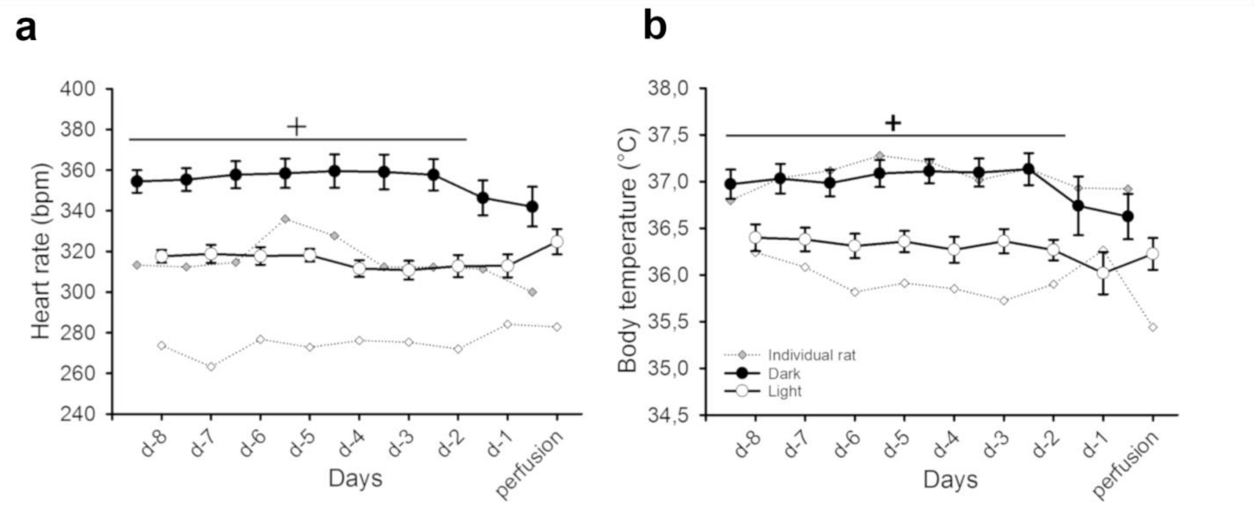

Suppl. Figure 3: Heart rate (bpm) was shown to be significantly different between the dark and the light phase until day-2 ($F(1,48)=56.910$, $p<0.001$; left). Body temperature (°C) measured subcutaneously was found to be different between light and dark phase ($F(1,48)=150.241$, $p<0.001$; right). Data are shown for the central 8 hours of the dark and the light phase measured by the telemetric device as mean ± S.E.M. Absolut values of one individual animal is shown with grey symbols.
